# On the Virtues of “Team Medicine”—A City of Hope Perspective

**DOI:** 10.3390/jcm12154897

**Published:** 2023-07-26

**Authors:** Prakash Kulkarni, Ravi Salgia

**Affiliations:** 1 Department of Medical Oncology, City of Hope National Medical Center, 1500 Duarte Rd., Duarte, CA 91010, USA; pkulkarni@coh.org; 2 Department of Systems Biology, City of Hope National Medical Center, 1500 Duarte Rd., Duarte, CA 91010, USA

Our first Special Issue of the *Journal of Clinical Medicine*, entitled ‘Integrating Clinical and Translational Research Networks—Building Team Medicine,’ highlighted the collective experience of the City of Hope and was a tremendous success. Buoyed by the enthusiastic response from our peers and colleagues, we embarked on bringing out the second Special Issue. The basic theme has remained the same, namely, integrating academic medical centers with their clinical network in various geographic locations to ensure that all patients, regardless of their physical proximity to major medical institutions, can benefit from recent clinical advances, and implementing exceptional care.

Like its predecessor, the collection of papers in this volume underscores the importance of integrating basic research scientists together with clinicians to enable a systems-level perspective. The two articles on addressing drug resistance by Kulkarni et al. [1] and Ramisetty et al. [2] provide examples of how this approach has uncovered new therapeutic strategies; they describe how the team medicine spirit helped address cisplatin resistance in NSCLC and identify a previously approved compound to alleviate cisplatin resistance in NSCLC, respectively.

Similarly, the article by Heater et al. [3] describes a new model utilizing online platforms to expand the reach of clinical expertise in the treatment of advanced soft-tissue sarcoma. Likewise, the paper by Sattler et al. [4] discusses the genetic/non-genetic duality in EGFR inhibitor drug resistance and underscores how team medicine approaches, wherein clinical developments work hand in hand with drug development research, drive potential opportunities for combination therapy.

For an expert clinical/research network with complementary expertise and the capability of multidisciplinary care, it is obvious that appropriate infrastructure would be necessary to empower this network to deliver personalized precision care to their patients. Thus, as cancer care becomes exponentially more complex with new diagnostic and therapeutic choices, providing decision support remains challenging. In an article offering a unique perspective, Bosserman et al. [5] describe how City of Hope has developed a pyramidal decision support framework to address these challenges, which have been exacerbated by the COVID pandemic, health plan restrictions, and growing geographic site diversity. They demonstrate how optimizing efficient and targeted decision support, backed by multidisciplinary cancer expertise, can improve individual patient treatment plans and thus achieve improved care and survival wherever patients are treated.

The paper by Mambetsariev et al. [6] discusses how cross-collaboration and integration between individual academic sites, national cancer networks, and community practices can enhance true personalized medicine. The implementation of these ideas, powered by recent advances in artificial intelligence and machine learning, could, in the future, allow for personalized high-throughput drug screenings that would yield faster drug discoveries and approved therapeutics. These are just a few examples highlighting the content of this thematic issue. Together, the 17 papers provide an overview of the state of the art of team medicine and its virtues in clinical practice.

With >125 faculty members in the Department of Medical Oncology, and 31 clinical network centers in Southern California, Arizona, Georgia, and Illinois, the City of Hope is an encompassing enterprise in which we have inculcated the team medicine philosophy in order to integrate basic and translational research, along with clinical medicine in academic centers and their clinical networks. Thus, we trust our colleagues across the US and around the world will find the approach described in the articles included herein—as well as those included in the predecessor volume [7]—useful for guiding their own approaches to treating cancer patients.
